# Regular exercise improves asthma control in adults: A randomized controlled trial

**DOI:** 10.1038/s41598-019-48484-8

**Published:** 2019-08-19

**Authors:** Jouni J. K. Jaakkola, Sirpa A. M. Aalto, Samu Hernberg, Simo-Pekka Kiihamäki, Maritta S. Jaakkola

**Affiliations:** 1 Center for Environmental and Respiratory Health Research (CERH), University of Oulu, Oulu, Finland; 2 Medical Research Center Oulu, Oulu University Hospital and University of Oulu, Oulu, Finland

**Keywords:** Clinical trials, Epidemiology

## Abstract

We conducted a randomized controlled trial to test the hypothesis that a 24-week exercise intervention improves asthma control in adults. Adults with mild or moderate asthma were randomly assigned to either the exercise intervention group (IG) or the reference group (RG). Participants in IG received an individualized exercising program, including aerobic exercise at least three times a week for ≥30 minutes, muscle training, and stretching. The primary outcome was asthma control, measured by Asthma Control Test (ACT), asthma-related symptoms, and peak expiratory flow (PEF) variability. We estimated the risk (i.e. probability) of improvement in asthma control and the risk difference (RD) between IG and RG. Of 131 subjects (67 IG/64 RG) entered, 105 subjects (51/54) completed the trial (80%), and 89 (44/45) were analysed (68%). The ACT became better among 26 (62%) participants in IG and among 17 (39%) participants in RG. The effect of intervention on improving asthma control was 23% (RD = 0.23, 95% CI 0.027–0.438; *P* = *0.0320*). The intervention also reduced shortness of breath by 30.1% (RD = 0.301, 95% CI 0.109–0.492; *P* = *0.003*). The change in PEF variability was similar in both groups. Regular exercise improves asthma control measured by the ACT, while has little effect on PEF variability.

## Introduction

The World Health Organization has estimated that 235–250 million people worldwide are affected by asthma^1^. Approximately 4.3% of the global adult population have asthma^2^. In spite of advancements in pharmacological treatment, poor asthma control continues to lead to a substantial number of health care visits and hospitalizations, and to lower quality of life.

Asthma is a chronic inflammatory disease of the airways, characterized by airway obstruction and bronchial hyperresponsiveness (BHR). The most common symptoms of asthma include cough, wheezing, shortness of breath/breathlessness, and chest tightness^3^. Aerobic exercise often provokes asthma-related symptoms, and may, therefore, lead to an aversion to physical exercise among asthmatics^4^. Previously, subjects with asthma were advised by their doctor to avoid exercise. However, regular aerobic exercise has been shown to produce substantial health benefits among healthy subjects. Because of these findings, more recently an interest has arisen on whether exercise might also be beneficial for asthma patients. Our previous systematic review and meta-analysis provided evidence that regular physical exercise improves physical fitness among adult asthmatics, but the evidence was insufficient to assess the effects of exercise on asthma control and quality of life^5^. To fill in this gap in knowledge, we conducted The Regular Exercise and Asthma Control Trial (REACT) to test the hypothesis that regular exercise improves asthma control.

## Results

### Participants

**Figure 1:**
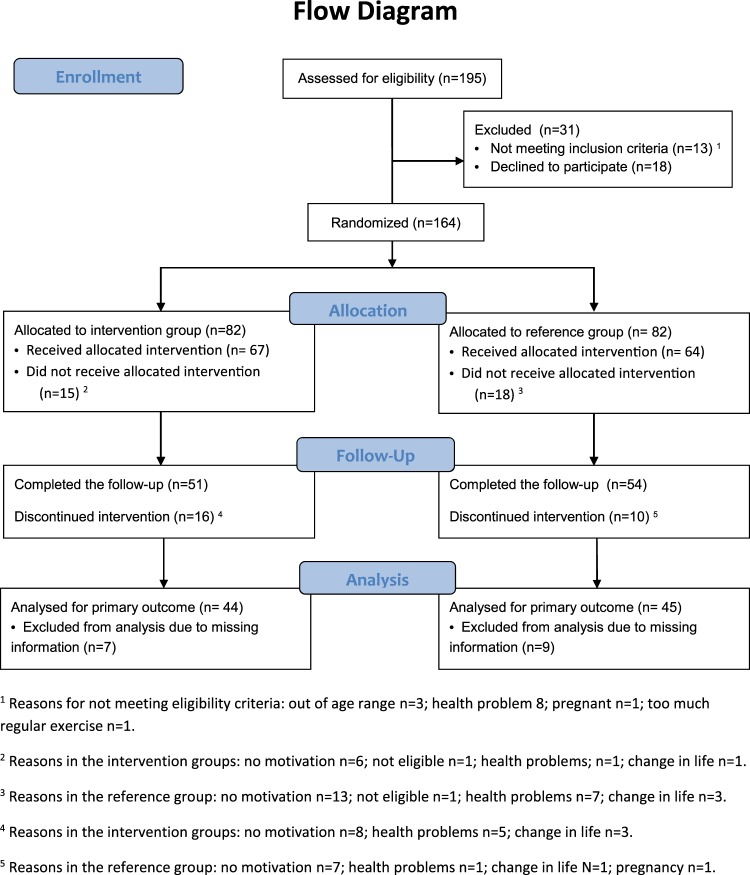
Flow diagram of the Regular Exercise and Asthma Control Trial (REACT).

We assessed 195 subjects for eligibility (see the criteria in Table 1); 13 subjects were not eligible and 18 declined to participate after the introduction to the study (Fig. 1). The remaining 164 subjects were randomly allocated into the intervention group (n = 82) and the reference group (n = 82). They received detailed information on the study and were asked to perform a step test. Additional 33 subjects were disqualified in the run-in phase. The remaining 131 subjects received the allocated active (n = 67) or reference intervention (n = 64). The entry time to the intervention was defined by the performance of the first spiroergometry testing. These two groups were broadly similar at the baseline, including the amount of regular exercise performed, the mean level of asthma control and experiencing asthma-related symptoms and use of asthma medication (Table 2). The mean age was significantly different between the intervention (mean 39.7, SD 14.06) and reference groups (mean 32.9, SD 10.72). A total of 51 (76%) subjects in the intervention group and 54 (84%) subjects in the reference group were followed for 6 months. The mean amount of exercise during the study period was 4.60 hours per week (SD 3.12) in IG and 3.39 hours per week (SD 3.03) in RG (*P* = *0.049*).

**Table 1:** Eligibility criteria, The Regular Exercise and Asthma Control Trial (REACT).

**Inclusion criteria**
Age range from 16 to 65 years
Diagnosis of asthma made by a physician and/or the reimbursement right for asthma medication from the Finnish National Social Insurance Institution (i.e. code 203)
In the case of newly diagnosed asthma, he/she had to fulfil the diagnostic criteria for asthma as outlined in the Finnish Guidelines for Asthma Management (a)
Mild or moderate asthma.
**Exclusion criteria**
Exclusion criteria for severe asthma at baseline included: (1) FEV1 < 60% of predicted in spirometry; (2) PEF variability >30% at least 2 times during an 1-week monitoring period; (3) use of short-acting bronchodilating medication at least four times daily; and/or (4) permanent daily oral steroid treatment.
The following chronic diseases: serious coronary heart disease, severe hypertension, severe heart failure, severe musculoskeletal disorder, dementia, and/or physician diagnosed chronic obstructive lung disease.
Exercising regularly at baseline (before the intervention) at least three times per week

^a^Duodecim. 2012. The Finnish Guidelines for Asthma Management (http://www.kaypahoito.fi/web/kh/suositukset/suositus?id=hoi06030, Accessed 20 July 2018).

**Table 2:** Baseline characteristics of the 89 participants analysed, The Regular Exercise and Asthma Control Trial [REACT].

Total	Intervention group	Reference group	Difference between the groups
	Analysed	Analysed	
	N = 44	N = 45	Test statistic, *P*-value
**Age**, Mean (SD)	39.7 (14.06)	32.9 (10.72)	t value = −2.56, 0.012
**Age group**			
18–29	12 (27.3)	23 (51.1)	Chi-square (d.f. = 2) = 7.5521 0.023
30–49	20 (45.5)	18 (40.0)	
50+	12 (27.3)	4 (8.9)	
**Gender**			
Men	8 (18.2)	11 (24.4)	Chi-square (d.f. = 1) = 0.5197 0.471
Women	36 (81.8)	34 (75.6)	
**Smoking**			
Never	34 (77.3)	32 (73.8)	Chi-square (d.f. = 2) = 0.8677 0.648
Ex	4 (9.1)	7 (15.6)	
Current	6 (13.6)	6 (13.3)	
**BMI**, Mean (SD)	24.97 (3.81)	24.95 (4.54)	t value = −0.02, 0.983
−18	1 (2.3)	0 (0.0)	Chi-square (d.f. = 3) = 1.1875 0.756
18–25	26 (59.1)	28 (62.2)	
25–30	10 (22.7)	11 (24.4)	
30−	7 (15.9)	6 (13.3)	
**6-min step test**, Mean, SD	200.3 (40.19)	202.5 (40.12)	t value = 0.25, 0.801
**Regular exercise** h per week, Mean, SD	3.99 (3.16)	3.33 (3.69)	t value = −0.91, 0.367
**ACT**, Median, 25,75%	21 [18, 22]	22 [19, 23]	Wilcoxon S = 1785.0, 0.107
20–25	29 (65.9)	33 (73.3)	Chi-square (d.f. = 1) = 0.394 0.5302
5–19	15 (34.1)	12 (26.7)	
**Shortness of breath weekly**	26 (59.1)	22 (48.9)	Chi-square (d.f. = 1) = 0.9320 0.334
**Shortness of breath less than weekly**	18 (40.9)	23 (51.1)	
**Wheezing weekly**	17 (39.5)	10 (22.2)	Chi-square (d.f. = 1) = 3.0988 0.078
**Wheezing less than weekly**	26 (60.5)	35 (77.8)	
**Phlegm weekly**	25 (58.1)	13 (28.9)	Chi-square (d.f. = 1) = 7.6680 0.006
**Phlegm less than weekly**	18 (41.9)	32 (71.1)	
**Coughing weekly**	16 (37.2)	15 (33.3)	Chi-square (d.f. = 1) = 0.1448 0.704
**Coughing less than weekly**	27 (62.8)	30 (66.7)	
**PEF** % predicted	104.92 (16.94)	108.53 (17.14)	F-test value = 1.00, 0.321
**FEV1**% predicted	92.27 (9.93)	94.53 (12.59)	F-test value = 0.88, 0.351
**FVC** % predicted	99.50 (8.13)	101.84 (12.77)	F-test value = 1.06, 0.306
**FEV1/FVC** % predicted	92.97 (9.21)	93.03 (7.31)	F-test value = 0.00, 0.969
**Short-acting bronchodilator**			
Regularly	4 (9.1)	1 (2.2)	
If needed	35 (79.6)	36 (80.0)	Chi-square (d.f. = 2) = 2.50,
Not at all	5 (11.4)	8 (17.8)	0.287
**Inhaled steroid**			
Regularly	24 (54.5)	26 (57.8)	
If needed	5 (11.4)	5 (11.1)	
Not at all	14 (31.8)	14 (31.1)	Chi-square (d.f. = 2) = 0.03,
Missing	1 (2.3)	0 (0.0)	0.983
**Oral steroids**			
Regularly	0	0 (0.0)	
If needed	6 (13.6)	12 (26.7)	Chi-square (d.f. = 1) = 2.18,
Not at all	37 (84.1)	33 (73.3)	0.139
Missing	1 (2.3)	0 (0.0)	

### Effects of intervention on asthma control

Of the 105 participants completing the trial, 89 (44 IG/45 RG) provided sufficient information for the analyses. In the IG, ACT score became better among 26 (62%) participants. In the reference group, 17 (39%) participants became better. Thus the effect of intervention assessed by the risk difference (RD) for better asthma control between intervention and reference groups, was 0.233 (95% CI 0.027–0.438; *P* = *0.032*), as shown in Table 3. The RD adjusted for gender, age, smoking, BMI, regular exercise at baseline, and baseline ACT (0.256, 95% CI 0.048–0.463; *P* = *0.016*) applying generalized linear model was similar and statistically significant. In addition, the intervention reduced significantly the use of asthma rescue medication, with a RD of 0.239 (95% CI 0.063–0.414, *P* = *0.001*) (Table 3).

**Table 3:** The effects of the 6-month exercise intervention on asthma control.

Outcome measure, past 4 weeks	Intervention group (N=44)				Reference group (N=45)				Effect of intervention: RD (95% CI)	Hypothesis testing: P for RD=0
	Tot N	Better n Risk b	No change n	Worse n Risk w	Total N	Better n Risk b	No change n	Worse n Risk w		
*Asthma Control Test*	42	26	7	9	44	17	17	10	0.233	0.032
		0.619		0.214		0.386		0.227	(0.027–0.438)	
Getting things done at work school or both^a^	43	9	26	8	45	5	36	4	0.096	0.227
		0.209		0.186		0.133		0.089	(−0.058–0.249)	
Having shortness of breath^b^	42	11	26	5	45	13	24	8	−0.027	0.779
		0.262		0.119		0.289		0.178	(−0.215–0.161)	
Wake up at night due to asthma symptoms^c^	43	8	32	3	45	7	36	2	0.031	0.705
		0.186		0.070		0.156		0.044	(−0.127–0.188)	
Reduced use of rescue inhaler or nebulizer^d^	43	16	20	7	44	6	32	6	0.239	0.010
		0.372		0.163		0.136		0.136	(0.063–0.414)	
Self-rating of asthma control^e^	43	16	22	5	45	12	25	8	0.105	0.291
		0.372		0.116		0.267		0.178	(−0.088–0.299)	

Risk Differences (RD) estimated for the probability of improvement. The Regular Exercise and Asthma Control Trial [REACT].

^a^In the past 4 weeks, how much of the time did your asthma keep you from getting as much done at work, school or at home? All of the time (1 point); Most of the time (2); Some of the time (3); A little of the time (4); None of the time (5 points).

^b^During the past 4 weeks, how often have you had shortness of breath? More than once a day (1); Once a day (2); 3 to 6 times a week (3); Once or twice a week (4); Not at all (5).

^c^During the past 4 weeks, how often did your asthma symptoms (wheezing, coughing, shortness of breath, chest tightness or pain) wake you up at night or earlier than usual in the morning? 4 or more nights a week (1); 2 to 3 nights a week (2); Once a week (3); Once or twice (4); Not at all (5).

^d^During the past 4 weeks, how often have you used your rescue inhaler or nebulizer medication (such as salbutamol)? 3 or more times per day (1); 1 or 2 times per day (2); 2 or 3 times per week (3); Once a week or less (4); Not at all (5).

^e^How would you rate your asthma control during the past 4 weeks? Not controlled at all (1); Poorly controlled (2); Somewhat controlled (3); Well controlled (4); Completely controlled (5).

A total of 89 participants were included in some analysis. Individual analyses included from 86 to 88 participants.

The results from the predefined subgroup analyses by age, gender, and smoking, applying Mantel-Haenszel (MH) summary effect estimates, are shown in Supplementary Information Tables S1–S3. There were some differences in stratum-specific RD’s. The effect estimate for the younger stratum was substantially greater with an RD of 0.332 (95% CI 0.059–0.624, *P* = *0.025*) compared to that of the older group with an RD of 0.069 (−0.231–0.370, *P* = *0.653*). RD was elevated among women (0.294, 95% CI 0.067–0.521, *P* = *0.015*), whereas there was no evidence of effect among men (RD −0.025, 95% CI −0.478–0.428, *P* = *0.916*). Further, there was evidence of a significant effect among never smokers (RD = 0.334, 95% CI 0.101–0.566, *P* = *0.009*), but not among current or previous smokers (RD = −0.089, 95% CI −0.450–0.373, *P* = *0.334*). As a response to the review, we conducted also a *post hoc* subgroup analysis according to baseline ACT (Table S4). The effect estimate was greater for subjects with a better asthma control at baseline (RD = 0.253, 95% CI 0.008–0.497) compared to those with poor asthma control (RD = 0.097, 95% CI −0.265–0.459). This heterogeneity could have been explained by chance (*P-value for homogeneity* = *0.485*).

As secondary analyses, we assessed the effect of intervention on the mean intra-individual change in ACT (ΔACT) (see Supplementary Information Tables S5 and S6). The overall effect of intervention was 0.74 points improvement in ACT score (95% CI: −0.31 to 1.78, *P* = *0.166*). After adjusting for gender, age, BMI, smoking, regular exercise, and baseline ACT, the effect was 0.65 points (95% CI −0.39–1.69, *P* = *0.217*). Table S5 shows that the effect was stronger among the young (1.74, 0.60–2.28, *P* = *0.004*), normal weight (1.27, −0.03–2.57, *P* = *0.055*), and subjects with less regular exercise at baseline (1.10, −0.86–3.06, *P* = *0.258*). There was substantial interaction between the intervention and age (*P* = *0.034* for interaction term), BMI (*P* = *0.097*), and regular exercise at baseline (*P* = *0.165*) and the effect of intervention on ΔACT was statistically significant when the interaction term was included in the model. In the full model with these three interaction terms, the effect of intervention was significant, showing 3.09 points improvement (0.52–5.66, *P* = *0.019*).

### Effects of intervention on asthma-related symptoms

Table 4 shows the effects of intervention on the occurrence of shortness of breath, wheezing, cough, and phlegm. Of the individual symptoms, the occurrence of shortness of breath was statistically significantly reduced: the risk difference (RD) for improvement between the intervention and reference groups was 0.301 (95% CI 0.109–0.492, *P* = *0*.003).

**Table 4:** The effects of exercise intervention on asthma control based on baseline and 6-month follow-up surveys.

Outcome measure, past 4 weeks1	Intervention group (N = 44)				Reference group (N = 45)				Effect of intervention: RD (95% CI)	Hypothesis testing: P for RD = 0
	Tot N	Better n Risk b	No change n	Worse n Risk w	Tot N	Better n Risk b	No change n	Worse n Risk w		
Shortness of breath	44	23	13	8	45	10	27	8	0.301	0.003
		0.523		0.182		0.222		0.178	(0.109–0.492)	
Wheezing	43	13	22	8	45	14	29	2	−0.009	0.929
		0.302		0.186		0.311		0.044	(−0.202–0.184)	
Cough	43	13	26	4	45	10	31	4	0.080	0.395
		0.302		0.093		0.222		0.089	(−0.103–0.263)	
Phlegm	42	9	30	3	45	9	29	7	0.014	0.870
		0.214		0.071		0.200		0.156	(−0.156–0.185)	

89 participants completed both baseline and 6-month survey. The Regular Exercise and Asthma Control Trial [REACT]. A total of 89 participants were included in some analysis. Individual analyses included from 87 to 88 participants.

### Effects of intervention on peak expiratory flow variability

The mean morning and evening PEFs in the intervention and reference groups at the baseline were similar, as shown in Table 1. A total of 43 (64%) participants in the intervention group and 49 (77%) participants in the reference group provided a sufficient number of PEF measurements both at the baseline and 3-month follow-up for assessment of the effect (Table 5). The effect estimates of intervention on CV of morning PEF measurements was 0.5% (95% CI −0.88–1.88) and for CV of evening PEF measurements 1.3% (95% CI −0.53–3.05). Both of the effect estimates indicate a small, but non-significant increase in the CV. In summary, the intervention effects on PEF variability could be explained by chance.

**Table 5:** Changes in variability of peak expiratory flow (PEF).

Group	Baseline			Change 0–3 months					Change 0–6 months				
	N	CV Mean (%)	SD	N	CV Mean (%)	SD	Mean of intraindividual differences 95% CI	Difference between groups (95% CI)	N	CV Mean (%)	SD	Mean of intraindividual differences 95% CI	Difference between groups (95% CI)
**CV of morning PEF**													
Intervention	49	3.94	2.74	43	3.94	3.45	0.17 [−0.97, 1.30]	—	27	3.55	2.38	0.02 [−0.99, 1.02]	—
Reference	55	3.89	2.10	49	3.66	3.81	−0.19 [−1.36, 0.99]	—	33	3.48	2.24	−0.48 [−1.46, 0.49]	—
**Effect of intervention**								*0.35* *[−1.27, 1.97]*					*0.50* *[−0.88, 1.88]*
**CV of evening PEF**													
Intervention	49	3.77	2.27	43	3.61	3.36	−0.12 [−1.31, 1.07]	—	28	3.23	2.91	−0.37 [−1.63, 0.89]	—
Reference	55	4.98	3.37	49	3.43	3.01	−1.59 [−2.78, −0.40]	—	33	3.36	2.20	−1.64 [−2.94, −0.34]	—
**Effect of intervention**								*1.47* *[−0.20, 3.14]*					*1.26* *[−0.53, 3.05]*

Coefficient of variation (%) calculated for up to 7 daily measurements per period, The Regular Exercise and Asthma Control Trial [REACT]. CV = coefficient of variation = SD/mean (%).

## Discussion

The results of our RCT indicate that a 24-week regular exercising program improves asthma control in adults, as assessed by the standardized Asthma Control Test (ACT)^6^. The intervention had on average a small increasing effect on PEF variability, which could be explained by chance. The *a priori* planned subgroup analyses indicated that the observed beneficial effects on ACT were predominantly present among young, women, and never smokers (see Supplementary Information Tables S1–S3). *Post hoc* subgroup analyses indicated beneficial effects also among normal weight subjects and those exercising less at the baseline.

ACT improved among a significantly larger percentage of participants in the intervention group (62%) compared with the reference group (39%). The use of asthma rescue medication was also reduced statistically significantly in the intervention group compared with the reference group.

The overall effects of the intervention on shortness of breath were clearly positive, showing a 30% improvement. There were no significant differences in the change in wheezing, cough or phlegm between the groups, nor in PEF variability.

We aimed at recruiting 200 subjects, but we were able to recruit and randomize slightly less, i.e. 131 eligible subjects (67 IG, 64 RG). The identification of eligible and willing subjects was slower than anticipated, which together with financial constraint lead to achieving only 66% of the target number. A total of 105 subjects completed the study, giving a follow-up rate of 80%. The analyses for the main outcome included 89 subjects (44 IG, 45 RG), because 16 subjects had missing information. In spite of the smaller study size than anticipated, we detected statistically significant beneficial effects of intervention on asthma control.

All of the participants had received the right for subsidized asthma medication from The Finnish Social Insurance Institution, which requires strict diagnostic criteria applied for asthma to warrant this subsidizing right.

The primary measure for asthma control was ACT score, which is based on subjective assessment of five items related to asthma control. The range of ACT varies from 5 to 25 points, 25 points representing the best and 5 points the poorest control. An important validity issue in the measurement of asthma control is that the ACT score is subjective, and the meaning of a unit change in the ACT score is more comparable with-in individuals than between individuals. Thus, in the primary assessment of the effect of exercise intervention we focused on intra-individual comparison of the ACT score before and after the intervention, which indicated whether the asthma control was better after the intervention compared to before. We argue that the most valid assessment of the effect of intervention is the plain comparison of the percentage of individuals who improved between IG and RG, which was expressed as risk difference. As secondary analyses, we also compared between the two groups the mean change in ACT score. The use of the group mean change in ACT score assumes that for example a 2 point improvement in participant A corresponds to a combination of 1 point improvement in participant B and participant C.

Schatz and colleagues^7^ defined a minimal important difference in ACT score at 3 points among individuals. Use of minimal important difference (MID) in the judgement for meaningful change in ACT score may be informative in clinical practice when following patients over time. In the assessment of the effects of intervention, it is not meaningful to set a general limit for MID, because the unit change in the subjective measures such as ACT score is not comparable between individuals. In the present analysis, any improvement in the outcome measure over time was considered meaningful and informative.

The groups compared in the analyses were broadly similar, but there were differences including a statistically significant difference in age distribution, the reference group being on average younger. According to the original study protocol, we conducted a sub-group analysis stratified by age, gender, and smoking, which showed that beneficial effects of intervention were mainly present among the younger, women and never smokers (Tables S1–S3). The assessment of the heterogeneity of stratum-specific effects was limited by the small number of subjects (*P* values from 0.122 to 0.217). Finally, the adjustment for gender, age, BMI, smoking, the amount of regular exercise at baseline and baseline ACT in generalized linear models did not influence substantially the effect of intervention on asthma control, which remained statistically significant.

When our trial was designed and initiated, there was only limited evidence on the effects of regular exercise on asthma control. Two systematic reviews and meta-analyses summarized the available evidence, and both underlined the need for RCTs to study the effects of exercise on asthma control^5,8^. During the conduct and reporting of our study, results from three smaller RCT’s studying potential effects of aerobic exercise on asthma control among adult asthmatics were published^9–11^. Table 6 summarizes these studies together with the present study.

**Table 6:** A summary of the randomized controlled trials on the effects of aerobic exercise on asthma control.

Author, year	City, Country	Design	Participants randomised	Intervention programme	Asthma control measures	Exercise intervention group	Reference group	Effect of exercise intervention
França-Pinto *et al*.^9^	São Paolo, Brazil	Parallel two-arm 12-week RCT	Outpatients, aged 20–59 yrs; moderate or severe persistent asthma: 22 in exercise intervention; 21 in reference group	Intensive aerobic training 2x per week under suprvision	Asthma Control Questionnaire score	Baseline score: 1.4 points (SD 1.2) Change: 0.2 95% CI: −0.3 to 0.5	Baseline score: 1.6 points (SD 0.9) Change: 0.1 95% CI: −2.1 to 0.5	0.2 points 95% CI: −0.3 to 0.7 *P* = *0.457*
Freitas *et al*.^10^	São Paolo, Brazil	Parallel two-arm 12-week RCT	Outpatients, aged 30–60 yrs; moderate or severe asthma; obese (BMI 35- < 40): 27 in exercise & weight loss; 28 in weight loss group;	Intensive aerobic training and muscle exercise 2x per week	Asthma Control Questionnaire score	69% of subjects improved Baseline score: 2.0 (95% CI: 1.4 to 2.7) Follow-up score: 1.1 (0.4 to 1.5) Change: 0.9 (*P < 0.001)*	36% subjects improved No change between baseline and follow-up score	Difference *P* = *0.03*
Toennesen *et al*.^11^	Copenhagen, Denmark	Parallel four-arm 8-week RCT	Normal weight adults, aged 18–65 yrs: 36 in exercise intervention; 38 in dietary intervention; 37 in exercise & dietary 38 in reference group	Intensive aerobic interval training 3x per week under supervision	Asthma Control Questionnaire score	*Exercise:* Baseline score: 1.7 (SD 0.6) Change: 0.7 *Exercise & diet:* 1.9 (SD 0.7) 0.9	Baseline score: 1.8 (SD 0.7) Change: 0.3	*Ex vs. Ref:* 0.4 points 95% CI: −0.01 to 0.78 *P* = *0.06* *Ex & diet vs. Ref:* 0.6 points 95% CI: 0.14 to 1.02 *P < 0.05*
Jaakkola *et al*. the present study	Oulu, Finland	Parallel two-arm 24-week RCT	Adults with mild/moderate asthma, aged 16–65 yrs; 67 in exercise intervention; 64 in reference group	Personalized aerobic training 3x per week, muscle training 2x per week and stretching before and after training	Asthma Control Test score	62% of subjects improved; Baseline score: Median 21 (IQR 18 to 22) Change: 1.19 points 95% CI: 0.36 to 2.02 *P* = *0.006*	39% of subjects improved Baseline score: Median 22 (IQR 20 to 23) Change: 0.45 points 95% CI: −0.22 to 1.12 *P* = *0.178*	23.3% 95% CI: 2.7 to 43.8 *P* = *0.032* Crude: 0.74 points 95% CI: −0.31 to 1.78 *P* = *0.166* Adjusted: 3.99 points 1.32 to 6.66 *P* = *0.004*

França-Pinto and colleagues^9^ conducted a RCT of 58 asthma patients in São Paulo, Brazil. The patients were randomized into a 12-week aerobic training group and reference group. Aerobic training was reported to reduce asthma exacerbations and improve quality of life measured by the Asthma Quality of Life Questionnaire, but there was only a small non-significant effect on asthma control measured by the Asthma Control Questionnaire (ACQ) score (0.2 points, 95% CI −0.3 to 0.7, *P* = *0.457*).

Freitas and colleagues^10^ conducted a RCT of 55 asthma patients who were participating in a weight-loss program in São Paulo, Brazil. The obese asthma patients were randomized into a 12-week aerobic and muscle strength training group undergoing nutrition (caloric restriction) and psychological therapies and a reference group with a general weight-loss program. The exercise intervention improved the ACQ score among 69% of subjects, whereas 36% of subjects improved in the weight-loss program only group. The ACQ score improved on average 0.9 by points (*P* < *0.001)* in the exercise intervention group, whereas no such effect was observed in the weight-loss only group.

Toennesen and colleagues^11^ conducted a RCT of 125 asthma patients in Copenhagen, Denmark. Non-obese adults were randomized into an 8-week exercise intervention group, diet group, an exercise and diet group and a reference group. The exercise intervention improved the ACQ score on average by 0.4 points (95% CI: −0.01 to 0.78, *P* = *0.06*).

These studies were smaller and had a shorter intervention period compared with the present study. Their interventions comprised intensive, supervised aerobic exercising, whereas the intervention of the present study included a personalized exercising program consisting of aerobic exercise, as well as muscle training and stretching. Our idea was that this kind of intervention could be applied easily in clinical practice. Our primary outcome was based on the comparison of the number of subjects whose asthma control improved, and the effect estimate was statistically significant, showing a 30% improvement. As a secondary outcome, we estimated the mean change in the intra-individual Asthma Control Test score. The intervention improved the ACT score by 0.74 points (95% CI: −0.31–1.78, *P* = *0.166*), which did not reach statistical significance. However, the a priori and post hoc subgroup analyses indicated stronger effects among young, normal weight, and non-smoking subjects as well as among those reporting less exercise at baseline (Table S5). The effect of intervention was stronger and statistically significant, 3.09 points (95% CI: 0.52–5.66, *P* = *0.019*) when including potential confounders and interaction terms in the full model (Table S6).

Three recent epidemiological studies have assessed associations between the amount of regular physical exercise or physical activity and measures of asthma control^12–14^. According to the World Health Organization, physical activity is defined as “any bodily movement produced by skeletal muscles that require energy expenditure”, whereas physical exercise “is a subcategory of physical activity that is planned, structured, repetitive, and aims to improve or maintain one or more components of physical fitness”^15^. Heikkinen and colleagues^12^ studied the relation between the amount of regular physical exercise and asthma control among 162 young adults with asthma recruited from the Espoo Cohort Study. Asthma control was assessed by the occurrence of asthma-related symptoms, including wheezing, shortness of breath, cough, and phlegm production during the past 12 months. These were less common among subjects who reported doing regularly moderate exercise. The association was strongest among overweight subjects. Russell and colleagues^13^ conducted a longitudinal analysis of the Bergen cohort, assessing the association of baseline physical activity with follow-up asthma, incident asthma and symptoms. Light, but not vigorous, physical activity predicted less follow-up asthma. Loponen and colleagues^14^ studied the association between daily physical activity and decline in lung function among 201 patients with adult-onset asthma over a period of 12 years after the asthma diagnosis. They found that the high physical activity group had slower annual FEV1 and FVC decline compared with the low physical activity group. Physical activity was measured at the follow-up only. Findings of all the three epidemiological studies are consistent with a beneficial effect of moderate physical exercise or activity on asthma control. However, assessment of the direction of causality between physical exercise or activity and level of asthma control is more difficult to judge in non-experimental epidemiological studies than in randomized controlled trials.

Different types of mechanisms have been proposed for beneficial effects of regular aerobic training on asthma. First, aerobic training improves cardiopulmonary fitness, which is shown by an increased maximal oxygen consumption^5^. We have previously suggested that improved oxygen uptake capacity together with increased threshold for getting breathlessness would help those with asthma in coping with their everyday life with a lower effort level. This would thus improve effort-benefit ratio, leaving more breathing reserves^5^.

Secondly, it has been proposed that exercise has short-term beneficial effects on airway smooth muscle^16^. This is consistent with the idea presented by the American College of Sports Medicine guidelines, which suggests that although exercise may be a trigger for airways obstruction among asthmatics, it is possible to build up tolerance to physical activity over time^17^.

Thirdly, animal models have suggested that exercise training reduces airway responsiveness^18^ and inflammation in mice^19^. In a Brazilian RCT^10^, a 12-week aerobic training program reduced both BHR and serum proinflammatory cytokines interleukin-6 (IL-6) and monocyte chemoattractant-1 (MCP-1), as well as reduced sputum eosonophils and fractional exhaled nitric oxide (FeNO) in asthma patients with more inflammation. There is evidence on the importance of IL-6 and MCP-1 in airways inflammation and BHR in asthma^20^. The reduction in FeNO suggests that aerobic exercise reduces airway inflammation.

In summary, there is increasing evidence suggesting plausible mechanisms, which could explain the beneficial effects of regular exercise on asthma control observed in our RCT. Our RCT provides evidence that 24 weeks of regular aerobic exercise with muscle training and stretching, improves asthma control measured by a broad spectrum of indicators, including reduction in use of rescue asthma medication, while it seems to have little effect on PEF variability. The *a priori* subgroup analyses suggest that the beneficial effects are most prominent among the young, women, and never smokers.

## Methods

### Trial design

The Regular Exercise and Asthma Control Trial (REACT) was a parallel 24-week randomized controlled trial (RCT) conducted in the City of Oulu, Finland (NCT02012400). The Ethics Committee of the Oulu University Hospital District approved the study protocol on 11 November 2009. The study was then registered into the Oulu University Hospital Research Registry maintained by the Ethics Committee of the Oulu University Hospital and into ClinicalTrials.gov on 30 May 2013. We followed ethical guidelines and obtained informed consent from all participants. The trial protocol is available at the Center for Environmental and Respiratory Health Research (CERH) website (http://www.oulu.fi/cerh/node/50741). We followed CONSORT guidelines in the reporting of results.

### Participants

To be included in the study, the person had (1) to be 16 to 65 years old; (2) to have the diagnosis of asthma made by a physician, and/or to have received the reimbursement right for asthma medication from the National Social Insurance Institution (i.e. diagnostic code 203), or, (3) in the case of newly diagnosed asthma, he/she had to fulfil the diagnostic criteria for asthma as outlined in the Finnish Guidelines for Asthma Management. Only mild or moderate asthmatics were included in this study. The exclusion criteria for severe asthma included: (1) FEV1 < 60% of predicted in spirometry; (2) PEF variability > 30% at least 2 times during a 1-week monitoring period; (3) use of short-acting bronchodilating medication at least four times daily; and/or (4) permanent daily oral steroid treatment. In addition, subjects were excluded, if they had any of the following chronic diseases: serious coronary heart disease, severe hypertension, severe heart failure, severe musculoskeletal disorder, dementia, and/or physician diagnosed chronic obstructive lung disease. In addition, regular exercise at baseline (before the intervention) at least three times per week lead to exclusion.

### Study setting

We enrolled adult asthma patients from the City of Oulu in Finland. The recruitment to the trial started on October 2012 at the Kaakkuri Health Care Center in Oulu and expanded to the Finnish Student Health Service (FSHS) at the University of Oulu. In January 2013, we started electronic recruitment targeted at the students and staff of the University of Oulu and Oulu University of Applied Sciences. The recruitment ended at the end of June 2015, and the last follow-up was conducted in January 2016. The research nurse contacted by telephone all the individuals who had expressed interest in the study. The subjects fulfilling the inclusion criteria were invited to the first research appointment, where they received general information on asthma self-management, and the purpose and content of the trial were explained. They were asked to give their informed consent and after that, to perform a 6-minute step test. They were then given instructions to fill in the 4-week daily diary and to perform peak expiratory flow (PEF) measurements for one week. They then underwent a 4-week run-in phase, during which their suitability and motivation to participate in the study was further assessed. Patients assessed as being eligible and motivated for the study were randomized to either the exercise intervention group (IG) or the reference group (RG). The first spiroergometry testing marked the entry to the trial and started the 6-month follow-up period. The recruitment and clinical examinations continued throughout the year over the study period, except during holiday seasons in July and December.

### Exercise intervention

The goal of the intervention was to encourage and guide physically inactive asthma patients to achieve a level of regular exercising, which was considered to produce health benefits in general. In addition, according to our hypothesis, it would improve asthma control. The baseline asthma control of the patients was measured with the baseline questionnaire, a one-week PEF-follow-up, and 4 weeks of filling in a diary on asthma symptoms and activity limitations due to asthma. The patients were asked to fill in a questionnaire which inquired about respiratory symptoms, questions including Asthma Control Test (ACT) (see Table 3, footnote), co-morbidities, health care use in the previous six months, and lifestyle and other factors, such as smoking, that may influence asthma control. The physical condition at the start of study period was measured by spiroergometric test^21^, muscle strength testing, and a 6-minute step test (6MST). In 6MST, the participants were asked to take as many steps as possible on the step board (height 22 cm) during six minutes. The Borg Scale from 6 (“no feeling of exertion”) to 20 (“I can’t do any more”) was used to rate perception of exertion during the 6MST^22^.

At the baseline, a trained research nurse gave oral and written instructions to the members of IG for a 24-week individualized exercising program, including aerobic exercise, muscle training, and stretching. Participants were instructed to carry out aerobic exercise at least three times a week for at least 30 minutes. They received individualized advice on different forms of aerobic exercising. Suggested forms of aerobic exercising included rapid walking, jogging, running, Nordic walking, skiing, cycling, team games, dancing, and exercising in the gym using for example weights, rowing machine or cross-trainer. The targeted heart-rate level was 70–80% of the maximal heart rate, which was measured in spiroergometry at baseline. The participants were instructed to perform interval training, if the exercise caused asthma symptoms. They were also instructed to moderate their training during cold weather (temperature below −10 °C). They were advised to avoid strenuous exercising during high levels of pollen to which they were sensitized. In addition, they were instructed to perform muscle exercises to strengthen abdominal, back, upper body and thigh muscles at least twice a week. Further, the participants received instructions for stretching before and after aerobic and muscle exercises. The instructions were based on the Finnish National Guidelines on Exercising that aim at sufficient amount of aerobic exercise and comprehensive muscular training. Members of RG were encouraged to follow the routine asthma control instructions.

During the 12-week follow-up visit, the research nurse encouraged the intervention group participants to continue exercising regularly and gave additional instructions when needed. All participants were asked to report the duration, intensity and form of exercising they had performed on daily basis in their diary. In addition, they were asked to report any potential asthma symptoms and activity limitations in their diary. They were also asked to perform PEF-measurements twice a day for a one-week period every four weeks.

At the end of the 24-week period, the baseline measurements, including spiroergometry, muscle strength testing, 6-minute step test, and questionnaire were repeated. After this period, the members of the RG also received a personalized exercising program.

### Outcomes

The primary outcome was asthma control, which was measured by (1) the Asthma Control Test (ACT); (2) self-reported respiratory symptoms; and (3) PEF-measurements.

We applied ACT as an overall measure of asthma control^6^. ACT has been validated and widely used as a measure of asthma control. ACT comprises the following 5 items based on questions with responses scored from 1 to 5 points: (1) shortness of breath; (2) personal rating of asthma control; (3) use of asthma rescue medication; (4) limitations at work/school due to asthma; and (5) waking up because of asthma symptoms. The sum of the five item points forms the ACT result, which ranges from 5 to 25 points, 25 representing the optimal asthma control. The sum of the scores allows asthma control to be categorized as follows: uncontrolled asthma (5–19 points), controlled asthma (20–24 points), and optimal disease control (25 points).

We also assessed separately the occurrence of four asthma-related symptoms, including shortness of breath, wheezing, cough, and phlegm production during the past 12 months, at both baseline and 6-month follow-up surveys.

PEF variability was assessed based on a one-week, twice a day PEF monitoring using Vitalograph asma-1™ electronic respiratory monitor. Measurement periods were performed every 4 weeks. In addition, daily asthma symptoms and activity limitations were observed and marked in the diary.

Physical fitness was assessed by the spiroergometry and muscle strength testing was performed at the beginning and in the end of the 24-week study period. Furthermore, a 6-minute step test was carried out three times during the study: at the baseline, at the 3-month follow-up, and at the 6-month follow-up. The quality of life was assessed by St. George’s Respiratory Questionnaire (SGRQ) at the beginning, at the midpoint and at the end of the follow-up period.

### Sample size

We aimed at 100 study subjects in both groups. With a potential spontaneous improvement in asthma control in 20% among the RG, this would have detected a 20% effect with the power of 0.88 and the *P* level of 0.05. Because of financial and logistic constraints, the sample size was reduced to 82 subjects per group.

### Randomization and blinding

After the informed content was signed, the research nurse randomized 164 eligible participants individually and independently from each other by drawing a lot indicating either the exercise intervention group (IG) or the reference group (RG). This was an open trial as the participants could not be blinded, because of the nature of the intervention.

### Assessment of asthma control

The individual’s asthma control before and after the intervention was measured primarily by the Asthma Control Test^6^, and secondarily by the occurrence of four asthma-related symptoms (shortness of breath, wheezing, cough, and phlegm production), as well as by a 7-day morning and evening PEF variation (coefficients of variation in %).

### Statistical methods

The effect of intervention was assessed primarily by comparing the individual-level improvement in asthma control, and group-level difference in the mean risk (probability) of improvement (% of subjects) between IG and RG. The secondary assessment was based on a comparison of the mean of intra-individual change in the ACT score between the 6-month follow-up and baseline score (ΔACT). All analyses were performed according to intervention group allocation regardless of the amount of regular exercise actually performed during the study period. Our primary hypothesis was that the number of subjects, whose asthma improves, is greater in IG compared to RG. We tested the hypothesis by calculating the risk difference (RD) for improvement between IG and RG. We calculated *P*-values testing RD = 0 and also the corresponding 95% confidence intervals. We also calculated adjusted RD applying generalized linear models including concurrently the six core covariates: sex, age, smoking, Body Mass Index (BMI), regular exercise at baseline, and baseline ACT.

We conducted subgroup analyses for three *a priori* defined characteristics, including age (18–34 years and 35–64 years), gender, and smoking (never and ever), by calculating Mantel-Haenszel (MH) summary effect estimates, and by estimating the homogeneity of the stratum-specific effect estimates. In the secondary analyses, we compared the means of intra-individual differences in ΔACT as well as CV in PEF (as %) between the baseline and follow-up measurements. We estimated the absolute difference in ΔACT. We estimated adjusted ΔACT by fitting the main effects model, models stratified by the core covariates, and models including interaction terms between intervention and afore mentioned covariates. The full model included the intervention variable, six covariates listed above, and interaction terms with P value < 0.20.

We calculated the intra-individual differences in coefficients of variation (CV in %) of PEF for PEF measurements over 7 days, separately for both morning and evening recordings. We estimated the effect of intervention by comparing the group means by t-test.

The multivariate analyses applied Poisson regression using identity link function. Analyses were carried out using the GENMOD-procedure in the SAS software (SAS 9.3, SAS Institute, Inc., Cary, North Carolina).

### Ethics approval

Oulu University Hospital Ethics Committee (EETTMK: 106/2009).

## Supplementary information

Supplementary Information
