# Supplementary material for: Regular exercise improves asthma control in adults: A randomized controlled trial
Source: Sci Rep. 2019 Aug 19;9:12088. doi: 10.1038/s41598-019-48484-8 (PMC6700123; doi:10.1038/s41598-019-48484-8)
Supplement: Supplementary file 1 — Supplementary Information [file 41598_2019_48484_MOESM1_ESM.docx]

**Regular exercise improves asthma control in adults: A randomized controlled trial**

Jouni J. K. Jaakkola, Sirpa A. M. Aalto, Samu Hernberg, Simo-Pekka Kiihamäki & Maritta S. Jaakkola

**Supplementary Information**

**Supplementary Information**

**Table S1.** The effects of the 6-month exercise intervention on asthma control s*tratified by age*. Risk Differences (RD) estimated for the risk (probability) of improvement.

|  |  | **Intervention group (N=44)** | | | | | **Reference group (N=45)** | | | | | **Effect of intervention:**  RD  (95% CI) | **Hypothesis testing:**  P for RD=0 |
| --- | --- | --- | --- | --- | --- | --- | --- | --- | --- | --- | --- | --- | --- |
|  | |  |  |  |  |  |  |  |  |  |  |  |  |
| **Outcome measure,**  **past 4 weeks** | | Tot  N | Better  n  Risk b | No change  n | Worse  n  Risk w |  | Total  N | Better  n (%) | No change  n | Worse  n  Risk w |  |  |  |
| *Asthma Control Test* | | 42 | 26 | 7 | 9 |  | 44 | 17 | 17 | 10 |  | 0.233 | 0.032 |
|  |  |  | 0.619 |  | 0.214 |  |  | 0.409 |  | 0.227 |  | (0.027 – 0.438) |  |
| Age 18 to 34 years | | 18 | 11 | 5 | 2 |  | 26 | 7 | 12 | 7 |  | 0.332 | 0.025 |
|  |  |  | 0.611 |  | 0.111 |  |  | 0.308 |  | 0.269 |  | (0.059 – 0.624) |  |
| Age 35 to 64 | | 24 | 15 | 2 | 7 |  | 18 | 10 | 5 | 3 |  | 0.069 | 0.653 |
|  |  |  | 0.625 |  | 0.292 |  |  | 0.556 |  | 0.167 |  | (-0.231 – 0.370) |  |
| Maentel-Haenszel-Summary EE | |  |  |  |  |  |  |  |  |  |  | 0.207  (-0.002 – 0.418) | 0.055 |
|  |  |  |  |  |  |  |  |  |  |  |  |  |  |
| P-value for homogeneity | |  |  |  |  |  |  |  |  |  |  | 0.194 |  |

**Table S2.** The effects of the 6-month exercise intervention on asthma control s*tratified by gender*. Risk Differences (RD) estimated for the risk (probability) of improvement.

|  |  | **Intervention group (N=44)** | | | | | **Reference group (N=45)** | | | | | **Effect of intervention:**  RD  (95% CI) | **Hypothesis testing:**  P for RD=0 |
| --- | --- | --- | --- | --- | --- | --- | --- | --- | --- | --- | --- | --- | --- |
|  | |  |  |  |  |  |  |  |  |  |  |  |  |
| **Outcome measure,**  **past 4 weeks** | | Tot  N | Better  n  Risk b | No change  n | Worse  n  Risk w |  | Total  N | Better  n (%) | No change  n | Worse  n  Risk w |  |  |  |
| *Asthma Control Test* | | 42 | 26 | 7 | 9 |  | 44 | 17 | 17 | 10 |  | 0.233 | 0.032 |
|  |  |  | 0.619 |  | 0.214 |  |  | 0.409 |  | 0.227 |  | (0.027 – 0.438) |  |
| Men | | 8 | 3 | 2 | 3 |  | 10 | 4 | 3 | 3 |  | -0.025 | 0.916 |
|  |  |  | 0.375 |  | 0.375 |  |  | 0.400 |  | 0.300 |  | (-0.478 – 0.428) |  |
| Women | | 34 | 23 | 5 | 6 |  | 34 | 13 | 14 | 7 |  | 0.294 | 0.015 |
|  |  |  | 0.676 |  | 0.176 |  |  | 0.412 |  | 0.206 |  | (0.067 – 0.521) |  |
| Maentel-Haenszel-Summary EE | |  |  |  |  |  |  |  |  |  |  | 0.228 | 0.035 |
|  |  |  |  |  |  |  |  |  |  |  |  | (0.021 – 0.435) |  |
| P-value for homogeneity | |  |  |  |  |  |  |  |  |  |  | 0.2165 |  |

**Table S3.** The effects of the 6-month exercise intervention on asthma control s*tratified by smoking*. Risk Differences (RD) estimated for the risk (probability) of improvement.

|  |  | **Intervention group (N=44)** | | | | | **Reference group (N=45)** | | | | | **Effect of intervention:**  P for RD=0;  RD  (95% CI) | **Hypothesis testing:**  P for RD=0 |
| --- | --- | --- | --- | --- | --- | --- | --- | --- | --- | --- | --- | --- | --- |
|  | |  |  |  |  | Net n better  Risk |  |  |  |  | Net n better  Risk |  |  |
| **Outcome measure,**  **past 4 weeks** | | Tot  N | Better  n  Risk b | No change  n | Worse  n  Risk w |  | Total  N | Better  n (%) | No change  n | Worse  n  Risk w |  |  |  |
| *Asthma Control Test* | | 42 | 26 | 7 | 9 | 17 | 44 | 17 | 17 | 10 | 7 | 0.233 | 0.032 |
|  |  |  | 0.619 |  | 0.214 | 0.405 |  | 0.409 |  | 0.227 | 0.159 | (0.027 – 0.438) |  |
| Never smokers | | 32 | 21 | 4 | 7 | 14 | 31 | 10 | 13 | 8 | 2 | 0.334 | 0.008 |
|  |  |  | 0.656 |  | 0.219 | 0.438 |  | 0.355 |  | 0.258 | 0.097 | (0.101 – 0.566) |  |
| Current or previous smokers | | 10 | 5 | 3 | 2 | 3 | 13 | 7 | 4 | 2 | 5 | -0.039 | 0.857 |
|  |  |  | 0.500 |  | 0.200 | 0.300 |  | 0.538 |  | 0.154 | 0.385 | (-0.450 – 0.373) |  |
| Maentel-Haenszel-Summary EE | |  |  |  |  |  |  |  |  |  |  | 0.235  (0.027 – 0.444) | 0.031 |
|  | |  |  |  |  |  |  |  |  |  |  |  |  |
| P-value for homogeneity | |  |  |  |  |  |  |  |  |  |  | P=0.122 |  |

**Table S4.** The effects of the 6-month exercise intervention on asthma control s*tratified by baseline ACT*. Risk Differences (RD) estimated for the risk (probability) of improvement.

|  |  | **Intervention group (N=44)** | | | | | **Reference group (N=45)** | | | | | **Effect of intervention:**  P for RD=0;  RD  (95% CI) | **Hypothesis testing:**  P for RD=0 |
| --- | --- | --- | --- | --- | --- | --- | --- | --- | --- | --- | --- | --- | --- |
|  | |  |  |  |  | Net n better  Risk |  |  |  |  | Net n better  Risk |  |  |
| **Outcome measure,**  **past 4 weeks** | | Tot  N | Better  n  Risk b | No change  n | Worse  n  Risk w |  | Total  N | Better  n (%) | No change  n | Worse  n  Risk w |  |  |  |
| *Asthma Control Test* | | 42 | 26 | 7 | 9 | 17 | 44 | 17 | 17 | 10 | 7 | 0.233 | 0.032 |
|  |  |  | 0.619 |  | 0.214 | 0.405 |  | 0.409 |  | 0.227 | 0.159 | (0.027 – 0.438) |  |
| ACT 5-19 | | 15 | 11 | 1 | 3 | 8 | 11 | 7 | 3 | 1 | 6 | 0.097 | 0.604 |
|  |  |  | 0.733 | 0.067 | 0.200 | 0.533 |  | 0.636 | 0.273 | 0.909 | 0.545 | (-0.265 – 0.459) |  |
| ACT 20-25 | | 27 | 15 | 6 | 6 | 9 | 33 | 10 | 14 | 9 | 1 | 0.253 | 0.050 |
|  |  |  | 0.556 | 0.222 | 0.222 | 0.33 |  | 0.303 | 0.424 | 0.273 | 0.030 | (0.008 – 0.497) |  |
| Maentel-Haenszel-Summary EE | |  |  |  |  |  |  |  |  |  |  | 0.206 | 0.053 |
|  | |  |  |  |  |  |  |  |  |  |  | (0.002 – 0.410) |  |
| P-value for homogeneity | |  |  |  |  |  |  |  |  |  |  | 0.485 |  |

**Table S5.** The effects of the 6-month exercise intervention on asthma control measured as the mean of intra-individual change in Asthma Control Test Score (ΔACT) (N=86). Adjusted effect estimates from **g**eneralized linear regression models.

|  | **Crude ΔACT (95% CI)** | **P value** | **Adjusted ΔACT* (95% CI)** | **P value** |
| --- | --- | --- | --- | --- |
| All | 0.74 (-0.31 – 1.78) | 0.166 | 0.65 (-0.39 – 1.69) | 0.217 |
| Stratified by gender |  |  |  |  |
| Men | 0.48 (-1.64 – 2.59) | 0.640 | 0.92 (-2.08 – 3.92) | 0.514 |
| Women | 0.76 (-0.45 – 1.98) | 0.215 | 0.45 (-0.73 – 1.62) | 0.448 |
| Stratified by age |  |  |  |  |
| <35 | 1.95 (0.85 – 3.06) | 0.001 | 1.74 (0.60 – 2.88) | 0.004 |
| >35 | -0.65 (-2.45 – 1.15) | 0.468 | -0.68 (-2.61 – 1.24) | 0.476 |
| Stratified by BMI |  |  |  |  |
| <25 | 1.52 (0.37 – 2.67) | 0.011 | 1.27 (-0.03 – 2.57) | 0.055 |
| >25 | -0.47 (-2.48 – 1.54) | 0.637 | -0.32 (-2.27 – 1.63) | 0.739 |
| Stratified by smoking |  |  |  |  |
| Non-smoker | 0.96 (-0.31 – 2.23) | 0.136 | 0.84 (-0.42 – 2.10) | 0.188 |
| Smoker | 0.18 (-1.81 – 2.16) | 0.855 | 0.02 (-1.88 – 1.93) | 0.981 |
| Stratified by exercise at baseline |  |  |  |  |
| <2h/week | 2.25 (0.23 – 4.26) | 0.030 | 1.10 (-0.86 – 3.06) | 0.258 |
| >2h/week | 0.15 (-1.07 – 1.38) | 0.803 | 0.16 (-1.10 – 1.41) | 0.800 |
| Stratified by baseline ACT |  |  |  |  |
| 5-19 | 0.47 (-2.14 – 3.08) | 0.715 | -0.22 (-3.10 – 2.66) | 0.874 |
| 20-25 | 0.54 (-0.38 – 1.46) | 0.244 | 0.60 (-0.45 – 1.65) | 0.254 |

*Generalized linear models: Adjusted for gender, age, BMI, smoking, regular exercise at baseline, and baseline ACT.

**Table S6.** Influence of the inclusion of different interaction terms on the effect estimate, the mean of intra-individual change in Asthma Control Test Score (ΔACT) (N=86). The adjusted ΔACT from generalized linear regression models are presented with six interaction terms one at a time and with the three most important interaction terms together.

| **Interaction terms included** | **Adjusted ΔACT* (95% CI)** | **P value for the interaction** |
| --- | --- | --- |
| Main effects model | 0.65 (-0.39 – 1.69) |  |
| + Gender x Intervention | 0.44 (-0.74 – 1.62) | 0.461 |
| + Age x Intervention | 1.70 (0.30 – 3.10) | 0.034 |
| + BMI x Intervention | 1.37 (0.04 – 2.71) | 0.097 |
| + Smoking x Intervention | 0.71 (-0.49 – 1.91) | 0.826 |
| + Regular exercise x Intervention | 1.61 (-0.10 – 3.33) | 0.165 |
| + Baseline ACT x Intervention | 0.24 (-1.61 – 2.09) | 0.594 |
| + Age x Intervention,  + BMI x Intervention and  + Regular exercise x Intervention | 3.09 (0.52 – 5.66) | 0.103  0.251  0.279 |

*Generalized linear models: Adjusted for gender, age, BMI, smoking, regular exercise at baseline, and baseline ACT.
